# Induced expression and functional effects of aquaporin-1 in human leukocytes in sepsis

**DOI:** 10.1186/cc12893

**Published:** 2013-09-12

**Authors:** Alice G Vassiliou, Nikolaos A Maniatis, Stylianos E Orfanos, Zafeiria Mastora, Edison Jahaj, Triantafillos Paparountas, Apostolos Armaganidis, Charis Roussos, Vassilis Aidinis, Anastasia Kotanidou

**Affiliations:** 1 GP Livanos and M Simou Laboratories, First Department of Critical Care Medicine & Pulmonary Services, Medical School of Athens University, "Evangelismos" Hospital, 3 Ploutarchou Street, 10675 Athens, Greece; 2 Second Department of Critical Care, Medical School of Athens University, ‘Attikon’ Hospital 1, Rimini Street, 12462, Haidari, Athens, Greece; 3 First Department of Critical Care Medicine & Pulmonary Services, Medical School of Athens University, "Evangelismos" Hospital, 45-47 Ipsilantou Street, 10676 Athens, Greece; 4 Institute of Immunology, "Alexander Fleming" Biomedical Sciences Research Centre, 34 Alexander Fleming Street, 16672, Vari, Greece

## Abstract

**Introduction:**

Gene expression profiling was performed via DNA microarrays in leukocytes from critically ill trauma patients nonseptic upon admission to the ICU, who subsequently developed either sepsis (*n* = 2) or severe sepsis and acute respiratory distress syndrome (*n* = 3). By comparing our results with published expression profiling studies in animal models of sepsis and lung injury, we found aquaporin-1 to be differentially expressed across all studies. Our aim was to determine how the water channel aquaporin-1 is involved in regulating the immune response in critically ill patients during infection acquired in the ICU.

**Methods:**

Following the results of the initial genetic screening study, we prospectively followed aquaporin-1 leukocyte expression patterns in patients with ICU-acquired sepsis who subsequently developed septic shock (*n* = 16) versus critically ill patients who were discharged without developing sepsis (*n* = 13). We additionally determined aquaporin-1 expression upon lipopolysaccharide (LPS) exposure and explored functional effects of aquaporin-1 induction in polymorphonuclear granulocytes (PMNs).

**Results:**

Leukocyte aquaporin-1 expression was induced at the onset of sepsis (median 1.71-fold increase; interquartile range: 0.99 to 2.42, *P* = 0.012 from baseline) and was further increased upon septic shock (median 3.00-fold increase; interquartile range: 1.20 to 5.40, *P* = 0.023 from sepsis, Wilcoxon signed-rank test); no difference was observed between baseline and discharge in patients who did not develop sepsis. Stimulation of PMNs by LPS led to increased expression of aquaporin-1 *in vitro*, which could be abrogated by the NF-κB inhibitor EF-24. PMN hypotonic challenge resulted in a transient increase of the relative cell volume, which returned to baseline after 600 seconds, while incubation in the presence of LPS resulted in persistently increased cell volume. The latter could be abolished by blocking aquaporin-1 with mercury and restored by incubation in β-mercaptoethanol, which abrogated the action of mercury inhibition.

**Conclusions:**

Aquaporin-1 is induced in leukocytes of patients with ICU-acquired sepsis and exhibits higher expression in septic shock. This phenomenon may be due to LPS-triggered NF-κB activation that can also lead to alterations in plasma membrane permeability.

## Introduction

Septic shock, the most severe stage of the septic process, is a deadly disease defined as severe sepsis with hypotension that persists after resuscitation with intravenous fluid [1,2]. Critically ill patients may display a state of functional immune system suppression, which predisposes them to infections with opportunistic bacteria found in the ICU environment [3] and results in excess morbidity and mortality.

To identify genes and/or cellular pathways involved in the pathogenesis of ICU-acquired sepsis, we initially undertook leukocyte genomic expression analysis of polytrauma patients who developed severe sepsis during their ICU stay as compared with trauma patients who suffered from sepsis not escalating to a higher septic stage [2,4]. Following robust statistical selection of differentially expressed genes, results were compared with publicly available microarray datasets, thus creating a unique list of likely disease modifiers. Among these genes, aquaporin-1 (Aqp1), a water channel protein [5,6], was one of the three genes found deregulated in all datasets.

Aqp1, a 28 kDa integral membrane protein [7], exists as a tetramer with intracellular N-termini and C-termini – an organization similar to several ion channel proteins [8]. The functional significance of Aqp1 in migratory cells lies presumably in cell volume regulation and cytokinesis [9,10]. Aquaporin-9, an isoform of Aqp1, is constitutively expressed and regulates water influx at the leading edge of migrating neutrophils [11]. However, the role of Aqp1 in the molecular events taking place after leukocyte activation by pathogens remains unclear.

Based on the findings of the genomic expression analysis of our initial polytrauma patient group, we hypothesized that Aqp1 expression is induced in leukocytes of critically ill patients afflicted with nosocomial sepsis in a manner dependent on sepsis severity. To address this hypothesis and to study potential underlying mechanisms, we measured levels of leukocyte Aqp1 expression in critically ill patients at ICU admission, upon diagnosis of sepsis and at occurrence of septic shock; in additional *in vitro* experiments we probed the capacity of lipopolysaccharide (LPS) to regulate Aqp1 expression in polymorphonuclear granulocytes (PMNs) and investigated potential signaling pathways and functions.

## Materials and methods

The study was approved by the Evangelismos Hospital Research Ethics Committee and all procedures carried out on patients were in compliance with the Helsinki Declaration. Informed written consent was obtained from all patients’ next-of–kin prior to any study procedure.

### Gene expression profiling study population

We initially screened consecutive admissions of polytrauma patients to the Evangelismos Hospital ICU, Athens, Greece. Exclusion criteria were: no consent to participate, sepsis upon ICU admission, body mass index >35 kg/m^2^, age <18 years, pregnancy, brain death, end-stage cancer, total ICU stay <3 days, re-admission or transfer from another ICU, contagious diseases (human immunodeficiency virus, hepatitis), and oral intake of corticosteroids at an equivalent dosage ≥1 mg/kg prednisone/day for a period >1 month. The first five initially nonseptic patients (one female), who subsequently became septic were included in the study. Two blood samples were obtained per patient: the first upon admission to the ICU, and the second within 48 hours of sepsis. Total RNA was isolated and cRNAs were hybridized to Affymetrix Hu133A 2.0 GeneChips Affymetrix, Santa Clara, CA, USA according to the manufacturer’s instructions. This dataset has been deposited in ArrayExpress [ArrayExpress:E-MEXP-3621]. All methods employed in the aforementioned gene expression profiling study are described in detail in Additional file 1.

### Aqp1 gene expression in septic ICU patients: study population

Following the initial microarray study stated above, we sought to investigate Aqp1 leukocyte expression in critically ill adult, initially nonseptic, patients who were admitted to the multi-disciplinary ICU of Evangelismos Hospital and who subsequently developed sepsis and deteriorated to septic shock; an additional group of patients who did not develop sepsis during their stay in the ICU served as the control.

Prior to enrollment, we screened all consecutive admissions over a 12-month period for eligibility. Exclusion criteria were the same as those used for our initial gene expression profiling study population (above). Of the 118 subjects screened over the study period, finally 29 medical, surgical and trauma patients were enrolled in the study, 16 of whom developed septic shock and 13 who served as our control patients (did not develop sepsis during their stay in the ICU). Clinical data and blood samples were obtained from all patients enrolled. Patients were considered to have sepsis when they developed systemic inflammatory response syndrome as a result of documented infection, septic patients with evidence of organ dysfunction were considered to have severe sepsis, and septic patients with persisting hypotension (despite adequate fluid resuscitation) were considered to have septic shock, in accordance with international guidelines and recommendations [2,4].

Following study enrollment, baseline (upon ICU admission) anthropometric data and detailed organ system-oriented medical history were recorded. A venous blood sample was drawn at three different time points: at the time of ICU admission (baseline), at the onset of sepsis (within 6 to 12 hours) and upon septic shock. In patients who did not develop sepsis, blood samples were drawn at admission and at ICU discharge only. The age of the septic patients ranged from 21 to 82 years whereas the age of the patients that did not develop sepsis varied from 31 to 82 years. The characteristics of the abovementioned patients are presented in Table 1.

**Table 1:** Characteristics of the two ICU patient groups

**Characteristic**	**Sepsis/septic shock**	**Nonsepsis**	***P *** **value**
Number of patients	16	13	
Age (years)	47 ± 18	57 ± 17	0.12
Gender			
Male	13 (81.3%)	6 (46.2%)	0.06
Female	3 (18.7%)	7 (53.8%)	
Diagnosis			
Medical	5 (31.3%)	5 (38.5%)	0.71
Surgical or trauma	11 (68.7%)	8 (61.5%)	
ICU mortality	6 (37.5%)	0 (0%)	0.02
APACHE II score	15.3 ± 6.0	14.6 ± 5.0	0.76
SOFA score	8.3 ± 2.3	5.2 ± 1.8	<0.001
Sepsis day	4.2 ± 1.8	N/A	
Septic shock day	12.7 ± 10.0	N/A	

Data expressed as number (percentages) of total related variable and mean ± standard deviation. Student’s *t* test or the chi-square test was used, as appropriate. APACHE II and SOFA scores were estimated at ICU admission; sepsis and septic shock days denote the days post-ICU admission when sepsis and septic shock developed, respectively. APACHE, Acute Physiology and Chronic Health Evaluation; SOFA, Sequential Organ Failure Assessment.

### Chemicals and reagents

Whole blood was collected in Tempus Blood RNA tubes (Applied Biosystems, Life Technologies Corporation, Carlsbad, CA, USA). RNA extraction was performed with the Tempus spin RNA isolation kit (Applied Biosystems, Life Technologies Corporation). Taq DNA polymerase was purchased from Fermentas (Thermo Fisher Scientific Inc., Waltham, MA, USA), reverse transcriptase from New England Biolabs (Ipswich, MA, USA), the SYBR-Green assay from Finnzymes (Thermo Fisher Scientific, Waltham, MA, USA), and the nuclear extraction kit and the NF-κB (p65) transcription factor assay kit from Cayman Chemical Company (Ann Arbor, MI, USA). LPS, EF-24, dithiothreitol (DTT) and phenylmethylsulfonyl fluoride were from Sigma (Sigma-Aldrich Corp, St Louis, MO, USA). The PCR products were analyzed by agarose gel electrophoresis. All reagents used were of analytical grade.

### Blood collection

Three milliliters of venous blood were collected within the first 24 hours post ICU admission, at the onset of sepsis (within 6 to 12 hours) and upon septic shock or discharge from the ICU. Samples were collected in tubes containing RNA stabilizing solution (Applied Biosystems, Life Technologies Corporation).

### Total RNA extraction and evaluation of its quality

Total RNA was isolated from total blood cells (>95% leukocytes) using the Tempus spin RNA isolation kit (Applied Biosystems, Life Technologies Corporation) and following the manufacturer's instructions. Total RNA concentration and quality were determined spectrophotometrically at 260 and 280 nm, while RNA integrity was evaluated with formaldehyde agarose gel electrophoresis. Total RNA was stored at −80°C until use.

### Reverse transcription and PCR

One microgram of total RNA from each sample was reverse transcribed into single-stranded cDNA in a 20 μl reaction mixture, using the M-MuLV Reverse Transcriptase (New England Biolabs, Ipswich, MA, USA) and Oligo(dT) primers, following the manufacturer's instructions. The success of the synthesis of the single-stranded cDNA was tested by its PCR amplification. PCR was performed using 1.0 μl cDNA, 1.5 mM MgCl_2_, 400 μM dNTPs, 500 nM primers, 1.5 U Dream-Taq DNA polymerase and 1× reaction buffer (Fermentas, Thermo Fisher Scientific Inc.), on a PTC-200 thermocycler (MJ Research Inc., Waltham, MA, USA). Equal amounts (10 μl) of the 108 bp glyceraldehyde 3-phosphate dehydrogenase (GAPDH) and the 177 bp Aqp1 products were electrophoresed on a 2.5% agarose gel, visualized following ethidium bromide staining and photographed under ultraviolet light with a Kodak DC120 digital camera (Rochester, NY, USA).

### Quantitative real-time PCR

A highly sensitive quantitative real-time PCR method has been developed for the quantification of both GAPDH and Aqp1 mRNAs, with the use of SYBR® Green Dye detection systems. For the amplification of GAPDH (endogenous reference gene) as well as Aqp1 (target gene) mRNA sequences, gene-specific sets of primers were designed according to the information on the NCBI Sequence database and the advanced software of the Primer Express program. To avoid genomic DNA amplification, the primers, mentioned above, were chosen to span at least two exons. The sequences of the primers used were: for GAPDH, the forward 5′-ATGGGGAAGGTGAAGGTCG-3′ primer, consisting of 19 nucleotides, and the reverse 23-nucleotide 5′-TACATGAGGGCACGGAAGATG-3′ primer, which gave rise to a 108 bp amplicon; for the Aqp1 gene, the forward 5′-GGTGGGGAACAACCAGACG-3′ primer and the reverse 5′-TACATGAGGGCACGGAAGATG-3′ primer, each with a length of 23 nucleotides, produced an amplicon of 177 bp.

Quantitative real-time PCR analysis was performed in 96-well plates on a PTC-200 thermocycler (MJ Research Inc.). The 25 μl reaction mixture contained 5 ng cDNA, 300 nM primers and 1× DyNAmo SYBR® Green PCR Master Mix (Finnzymes, Thermo Fisher Scientific), in which modified Tbr DNA polymerase is included. The thermal protocol conditions consisted of 2 minutes at 50°C and 10 minutes at 95°C polymerase activation step, 40 cycles of denaturation at 95°C for 15 seconds, primer annealing at 54°C for 30 seconds, extension at 72°C for 10 seconds and a final extension step at 72°C for 10 minutes. All samples were amplified in triplicate and the average CT values were calculated for their subsequent expression analysis. Following amplification, a dissociation curve was generated to distinguish the PCR products of interest from the nonspecific ones or any primer dimers, through their particular melting temperatures (Tm), recorded in the software. Using the comparative 2^−ΔΔCT^ method [12] and the baseline samples as a calibrator, the relative quantification of the expression analysis of all blood samples was carried out. GAPDH expression was used for the normalization of Aqp1 expression levels between the different samples.

### Polymorphonuclear granulocyte isolation

PMNs were isolated from peripheral blood of consecutive healthy volunteers – blood donors supplied by the Blood Bank of Evangelismos Hospital. Blood was collected in 17% citric acid and allowed to sediment in 3% Dextran/0.9% NaCl for 1 hour. Leukocyte-rich supernatant was separated by centrifugation on Histopaque (Sigma, Sigma-Aldrich Corp.). After lysis of residual erythrocytes by hypotonic shock in ice-cold distilled water, the cells were resuspended in Hank's balanced salt solution (Gibco, Life Technologies Corporation, Carlsbad, CA, USA) and stored on ice until use. The cells were counted and diluted to the desired concentration.

### Culture of polymorphonuclear granulocytes

PMNs (2×10^6^) were cultured in Hank’s balanced salt solution using six-well plates. LPS from *Pseudomonas aeruginosa* 10 (Sigma, Sigma-Aldrich Corp.) was added at a final concentration of 100 ng/ml in the absence or presence of 1 μM NF-κB inhibitor EF-24 (Sigma, Sigma-Aldrich Corp.). The cells were incubated for 3 hours at 37°C. Following incubation, the cells were harvested for RNA and/or protein isolation. Each experiment was independently performed five times.

### RNA isolation from polymorphonuclear cells

RNA extraction was performed using the Trizol® reagent and the PureLink RNA Mini kit (Invitrogen, Life Technologies Corporation) following the manufacturer’s instructions. RNA was stored at −80°C until use. Reverse transcription and quantitative real-time PCR were performed as described above.

### Polymorphonuclear cell homogenate

Approximately 2×10^6^ cells were homogenized in PBS buffer with the addition of 5 μM DTT, 0.1 mM phenylmethylsulfonyl fluoride in isopropanol, and protease inhibitors, using a sonicator (Sonic Dismembrator 550; Fisher Scientific, Loughborough, UK) and 3×10 second bursts. The homogenate was centrifuged in a 5804 R Eppendorf centrifuge (rotor A-4-44; Eppendorf AG, Hamburg, Germany) for 5 minutes at 4°C and 1,000×*g*. The supernatant, referred to as total protein, was collected and stored at −80°C until use.

### Protein determination

Total protein concentration was determined according to the method of bicinchonic acid [13], using bovine serum albumin as standard.

### SDS-polyacrylamide gel electrophoresis

SDS-PAGE was performed on a Biorad Mini Protean II electrophoresis apparatus (Bio-Rad, Hercules, CA, USA), as described [14], using 12% polyacrylamide slab gels. Electrophoresis was carried out at 150 V for 1 hour at room temperature.

### Immunoblotting of polymorphonuclear homogenates with Aqp1 antibody

Following electrophoresis, samples were transferred onto an Immobilon-P polyvinylidene difluoride membrane (Millipore, 0.45 μl pore size; Millipore Corporation, Billerica, MA, USA). Western transfer was performed on a wet transfer apparatus (Bio-Rad). Immunological detection was performed according to the method of Batteiger and colleagues [15]. The polyclonal antibody against Aqp1 purchased from Santa Cruz Biotechnology, Inc. (Santa Cruz Biotechnology, Inc., Dallas, TX, USA) was used for the detection.

### Cell volume measurements

Osmotic swelling was monitored as in [16] with slight modifications. Briefly, swelling was monitored at 22°C with a Zeiss Axiovert 25 phase contrast microscope (Carl Zeiss AG, Oberkochen, Germany) with two 10× eyepieces and a 40× objective on a trinocular base, 0.5-numerical aperture long working distance Canon objective equipped with a Canon digital camera (Canon USA Inc., Melville, NY, USA). PMN were transferred from 300 mosM (in) to 150 mosM (out) Hank’s balanced salt solution diluted with water. PMN images were taken at 30-second intervals for a total of 600 seconds or until the time of PMN rupture. The relative volume V/Vo, that is the ratio of measured volume at various time-points during the osmotic challenge to the baseline volume, was determined. Changes in volume were measured by ImageJ software (NIH, Bethesda, MD, USA).

### Cytokine measurement

IL-8 levels were measured at the supernatants of the PMN cells by ELISA, according to the manufacturer’s instructions. The human IL-8 immunoassay of R&D Systems Inc. (Minneapolis, MN, USA) was used. This assay uses two different polyclonal antibodies against the cytokines as the catching antibody and the tagging antibody. Samples were assayed in triplicate.

### Polymorphonuclear cell nuclear extraction

Nuclear extraction was performed with the kit by Cayman Chemical Company, according to the manufacturer’s instructions. In brief, approximately 2×10^6^ cells were homogenized in pre-extraction buffer containing DTT and protease inhibitors. The cytoplasmic extract was removed from the nuclear pellet, which was treated further with extraction buffer containing DTT and protease inhibitors. The extract was incubated on ice for 15 minutes with vortex (5 seconds) every 3 minutes. The suspension was centrifuged in an Espresso centrifuge (Thermo Fisher Scientific Inc.) for 10 minutes at 9,800×*g* at 4°C and transferred into a new microcentrifuge vial. The supernatant, referred to as nuclear extract, was used for the measurement of NF-κB activation. Total protein concentration was determined by the bicinchonic acid method, as mentioned in the Protein determination section above.

### NF-κB transcription factor activation measurement

The activation of NF-κB transcription factor was measured for the nuclear extracts of PMN cells by the Transcription Factor Assay kit (Cayman Chemical Company), according to the manufacturer’s instructions. A specific double-stranded DNA sequence containing the NF-κB response element is immobilized onto the bottom of wells of a 96-well plate. NF-κB contained in a nuclear extract binds specifically to the NF-κB response element. NF-κB (p65) is detected by addition of specific primary antibody directed against NF-κB (p65). A secondary antibody conjugated to horseradish peroxidase is added to provide a sensitive colorimetric readout at 450 nm. Samples were assayed in triplicate.

### Statistical analysis

Data are presented as individual values, mean ± standard deviation for normally distributed variables and median with interquartile range for variables with skewed distribution. For continuous variables, the *t* test was used for two-group comparisons. Associations between qualitative variables were examined by the chi-square test. The Friedman test was used to examine differences within more than two groups, and the Wilcoxon signed-rank test for differences within two groups, one-way and two-way analyses of variance for repeated measures and Kruskal–Wallis analysis of variance followed by Newman–Keuls or Bonferroni’s *post-hoc* test were performed as appropriate. The Spearman’s rank correlation coefficient was calculated to measure the strength of relationship between levels of Aqp1 expression and time. All aforementioned analyses were performed using the Graphpad prism 5 statistical program (GraphPad Software, Inc., La Jolla, CA, USA). All *P* values are two-sided; *P* <0.05 was considered significant.

## Results

### Gene expression profiling in the initial polytrauma patients

Isolated RNA from total blood cells (>95% leukocytes) of the initial five polytrauma patients was hybridized to DNA microarrays (Affymetrix Hu133A 2.0) and genes with statistically significant changes in their expression in all patients were selected as described in detail in Additional file 1. Patients’ age was 21.75 ± 2.5 and mean ICU stay was 13 ± 5.2 days. Two of the patients developed sepsis while three developed severe sepsis and acute respiratory distress syndrome [2,4,17]. The two and three patient subgroups were analyzed independently (see Additional file 1) to detect genes that were differentially regulated between them, and which could be involved in pathogenetic mechanisms of the septic process and acute respiratory distress syndrome. We thus found 1,199 genes with statistically significant differences among the replicate values of fold-changes of the two separate patient subgroups (*P* < 0.05; false discovery rate 5%) (Additional file 1: Table S1).

To validate the selected genes, we compared our results with published expression profiling studies using animal models of LPS-induced and aseptic lung injury, as described in Additional file 1. The comparison analysis yielded three genes that were commonly identified as deregulated both in human patients with severe sepsis and acute respiratory distress syndrome, as well as experimental rodents: the orphan G-protein coupled receptor 182; the dedicator of cytokinesis protein 9, a Cdc42 guanine nucleotide exchange factor; and Aqp1, a 28 KDa water channel protein.

### Aqp1 gene expression in septic ICU patients

**Figure 1:**
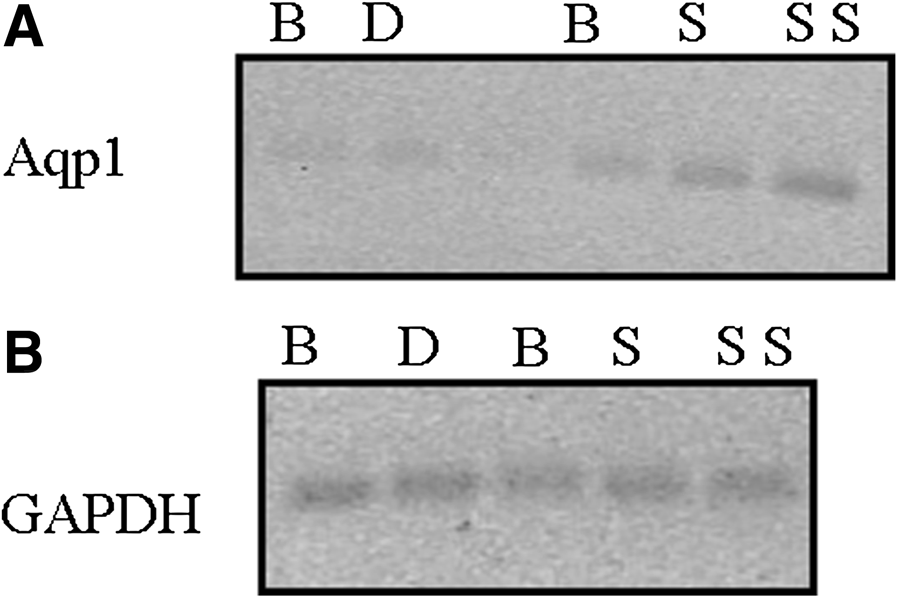
**Expression of aquaporin-1 in peripheral leukocytes of septic and nonseptic patients.** Representative expression analysis of aquaporin-1 (Aqp1) and glyceraldehyde 3-phosphate dehydrogenase (GAPDH) mRNAs in a nonseptic patient and a septic patient. End-point PCR was performed on RNA isolated from leukocytes of both patients at different time points (B, baseline; D, discharge; S, 6 to 12 hours from sepsis; SS, upon septic shock) and the end products were analyzed on a 2.5% agarose gel. **(A)** Expression of Aqp1 messenger RNA in peripheral leukocytes of one nonseptic patient and one septic patient. Lanes 1 and 2, nonseptic patient; lane 3, molecular weight standards; lanes 4 to 6, septic patient. **(B)** GAPDH was used as a positive control. Lanes 1 and 2, nonseptic patient; lanes 3 to 5, septic patient.

Since little is known regarding Aqp1 expression in leukocytes, we initially performed PCR on RNA isolated from leukocytes of ICU patients in order to confirm the findings of the gene expression analysis. Aqp1 expression was indeed detected in human leukocytes by end-point PCR (Figure 1A), in patients who either developed sepsis or did not. GAPDH was used as a positive control in the PCR reaction (Figure 1B).

**Figure 2:**
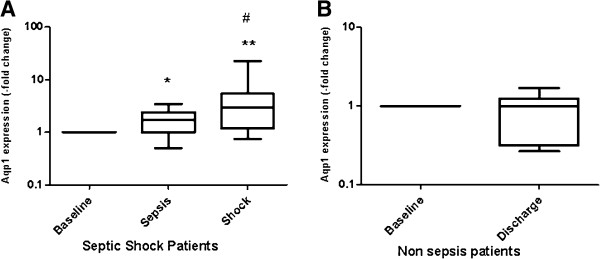
**Aquaporin-1 expression is induced in leukocytes of patients with sepsis. (A)** Distribution of aquaporin-1 (Aqp1) mRNA levels in septic shock patients at three different time points (baseline, 6 to 12 hours from sepsis and upon septic shock). **(B)** Distribution of Aqp1 mRNA levels in nonseptic patients at two different time points (baseline and discharge). In patients whose ICU stay was not complicated by sepsis, leukocyte Aqp1 levels at discharge did not exceed admission levels. In patients who developed sepsis, however, Aqp1 levels rose steadily from admission to the onset of sepsis and, up to the onset of septic shock. Data are presented as box plots. Line in the box, median value; box edges, 25th to 75th centiles; whiskers, range of values. *P <0.05, **P <0.01 from baseline values. #*P* <0.05 from sepsis. Differences within groups were tested by the Friedman test (more than two groups) and the Wilcoxon signed-rank test (two groups).

Following confirmation that Aqp1 is expressed in human leukocytes, we prospectively followed temporal trends in Aqp1 mRNA levels using quantitative real-time PCR in ICU patients who were initially nonseptic but later developed nosocomial sepsis and subsequently septic shock (Figure 2A); mRNA levels of ICU patients who did not develop sepsis, upon ICU admission and discharge, are shown in Figure 2B. All 16 patients who developed sepsis and then septic shock had positive biological fluid cultures, with 11 out of 16 having positive blood cultures. In one-half (8/16) of our patients, sepsis originated from lung infection; no difference in Aqp1 expression was noted in relation to the site of infection (data not shown).

Two of the 16 patients received short-term treatment (1 and 2 days, respectively) with short half-life steroids during their ICU stay. These regimens were far from the time points of Aqp1 expression measurements. Clinical characteristics of the patients are listed in Table 1. For each patient, Aqp1 levels on admission (baseline) are set as the reference value and the subsequent time points are expressed as the fold-change relative to this. Admission Aqp1 levels in ICU patients did not differ significantly from healthy blood donors (data not shown), nor did the baseline levels of patients who developed sepsis differ from the baseline levels of patients who did not develop sepsis (data not shown). However, upon onset of sepsis, we observed a median 1.7-fold (interquartile range: 0.99 to 2.4) increase in Aqp1 mRNA (*P* = 0.012) (Figure 2A). Aqp1 mRNA was also measured upon septic shock and was further elevated, corresponding to a median increase of 3.00-fold (interquartile range: 1.20 to 5.40, *P* = 0.002 from baseline and *P* = 0.023 from sepsis) (Figure 2A). Conversely, in patients who did not develop sepsis, Aqp1 expression at discharge did not differ from admission levels (median 0.99-fold, interquartile range: 0.32 to 1.24; Figure 2B). In the latter group, two additional patients also received short-term treatments of comparable duration with those of the septic subjects, with short half-life steroids. Spearman’s rank correlation coefficient revealed no statistically significant relationship between time (days) in the ICU and levels of Aqp1 expression in both groups.

### Aqp1 mRNA and protein expression in human PMNs stimulated by lipopolysaccharide

**Figure 3:**
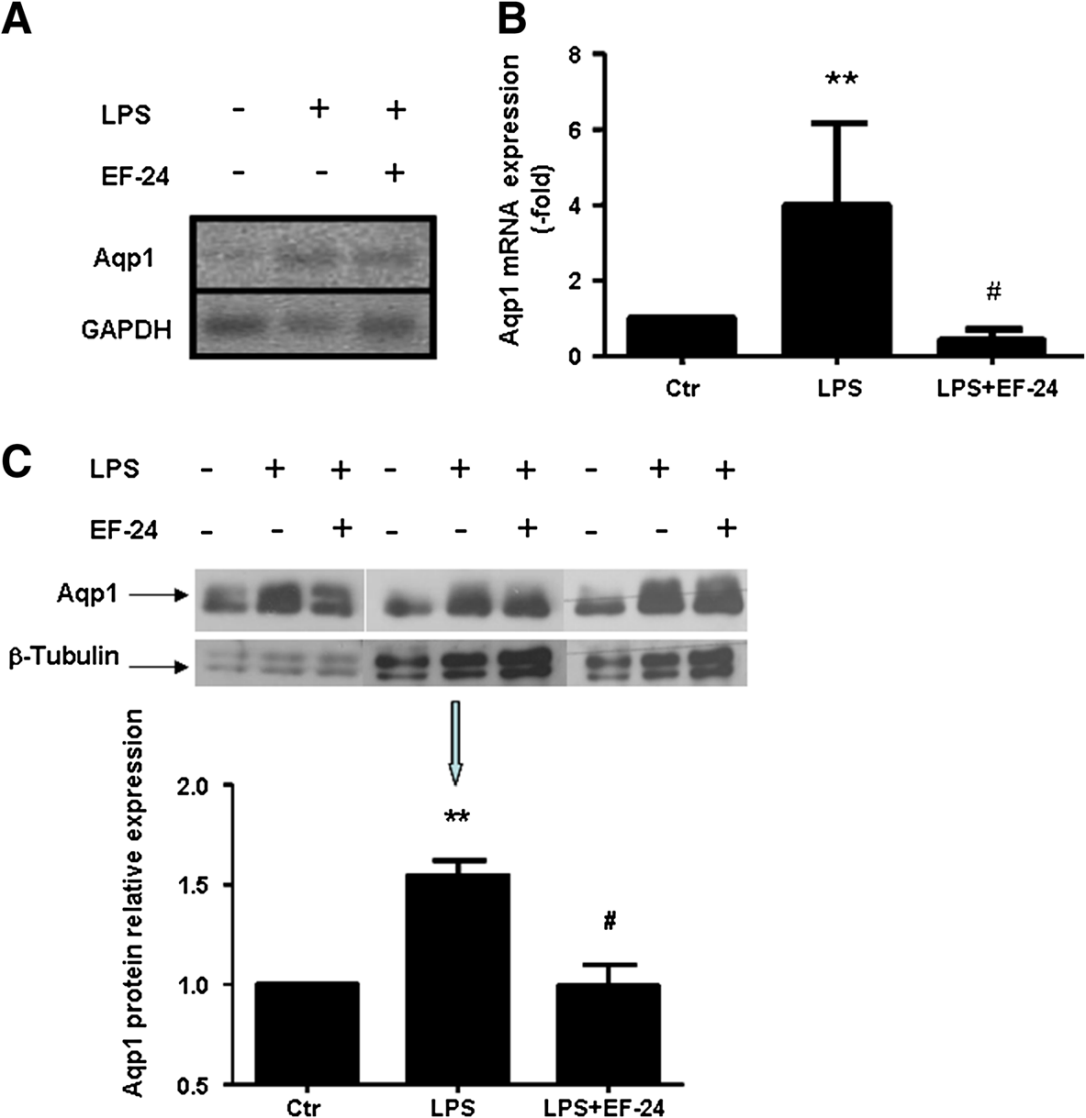
**Expression of aquaporin-1 mRNA and protein in polymorphonuclear granulocytes is induced by lipopolysaccharide.** Polymorphonuclear granulocytes (PMNs) isolated from healthy blood donor volunteers were incubated for 3 hours at 37°C in six-well plates with plain cell-culture medium (ctr) or lipopolysaccharide (LPS) from *Pseudomonas aeruginosa* in the absence or presence of NF-κB inhibitor EF-24. **(A)** Representative expression of aquaporin-1 (Aqp1; upper panel) and glyceraldehyde 3-phosphate dehydrogenase (GAPDH; lower panel) mRNAs. End-point PCR was performed on RNA isolated from PMNs, as above and analyzed on 2.5% agarose gel. **(B)** Fold-increase of Aqp1 gene expression, as measured by real-time PCR in PMNs. **(C)** Increase in protein expression of Aqp1 in crude protein samples isolated from PMNs, as analyzed by SDS-PAGE and immunoblotting. Relative Aqp1 protein expression was estimated by densitometry using β-tubulin as a loading control. **P <0.01 from corresponding control. #*P* <0.05 from corresponding LPS treatment by one-way analysis of variance followed by Newman–Keuls multiple comparison test.

Since Aqp1 appears upregulated in leukocytes from critically ill patients with nosocomial, mostly Gram-negative sepsis, we probed the role of LPS as an Aqp1 inducer. PMNs, particularly neutrophils, by virtue of their abundance and immediate responsiveness with genomic alterations to bacterial pathogens, became the focus of these studies. After confirming that Aqp1 is expressed in PMNs by end-point PCR (Figure 3A), healthy-donor PMNs were stimulated by LPS (100 ng/ml for 3 hours) *in vitro* and Aqp1 mRNA levels were determined by quantitative real-time PCR. In five independent repetitions of this experiment, Aqp1 mRNA expression rose by fourfold on average in the presence of LPS (*P* <0.01 compared with unstimulated neutrophils) (Figure 3B). This was also associated with an increase in Aqp1 protein levels in LPS-stimulated PMNs as detected by immunoblotting (Figure 3C).

**Figure 4:**
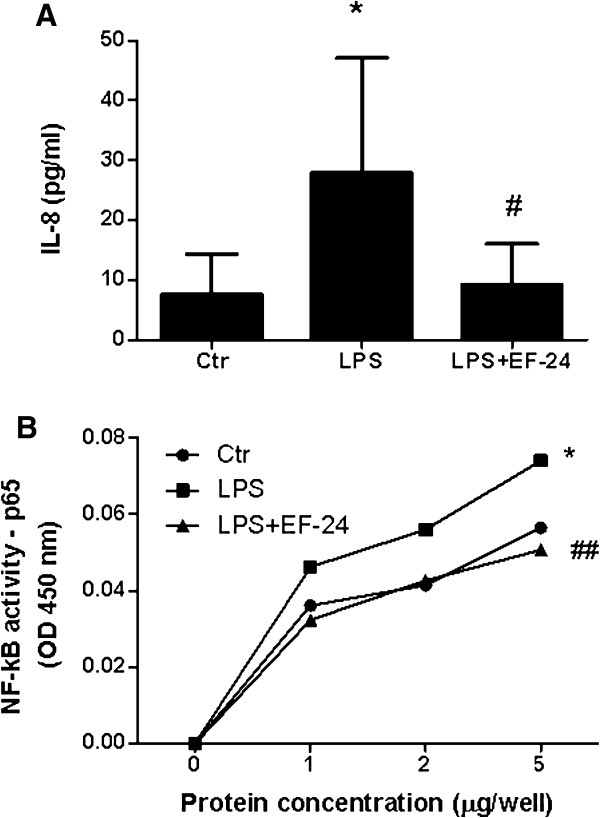
**Polymorphonuclear granulocytes are activated by lipopolysaccharide via NF-κB.** Polymorphonuclear granulocytes (PMNs) isolated from healthy blood donors were incubated for 3 hours at 37°C in six-well plates with plain cell-culture medium (ctr) or lipopolysaccharide (LPS) from *Pseudomonas aeruginosa* in the absence or presence of the NF-κB inhibitor EF-24. **(A)** IL-8 levels measured by ELISA on PMN supernatants. **(B)** NF-κB activation as measured by the NF-κB (p65) transcription factor assay on PMN nuclear extracts, expressed as increase of absorbance at 450 nm relative to total protein concentration. The increase in absorbance at 450 nm indicates translocation of NF-κB into the nucleus and DNA binding activity in the nuclear extracts. *P <0.05 from corresponding control. #*P* <0.05, ##*P* <0.01 from corresponding LPS treatment by one-way analysis of variance (ANOVA) followed by Newman–Keuls multiple comparison test or two-way ANOVA for repeated measures followed by Bonferoni’s *post-hoc* test.

Binding of LPS on Toll-like receptor-4 leads to NF-κB-mediated transcription of target genes, including IL-8; the latter was, in fact, found increased in cell culture supernatants in our experiments (Figure 4A), in support of NF-κB activation in our system (Figure 4B). Suppression of NF-κB activity by EF-24 (Figure 4B), through direct inhibition of IκB kinase, prevented Aqp1 mRNA induction (Figure 3B) and protein expression induced by LPS (Figure 3C), and IL-8 release (Figure 4A).

### Cell volume measurements

**Figure 5:**
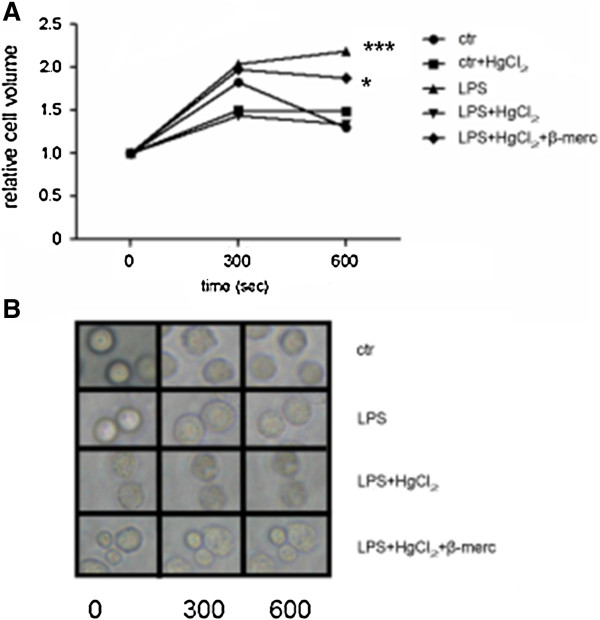
**Time-course and Hg**^**2+ **^**inhibition of osmotic swelling of polymorphonuclear granulocytes. (A)** Time course and Hg^2+^ inhibition of osmotic swelling of polymorphonuclear granulocytes (PMNs) that were incubated for 3 hours at 37°C either in the absence (control) or presence of lipopolysaccharide (LPS). Cells were then transferred to a hypotonic solution and changes in size were measured by videomicroscopy (see Materials and methods). Where indicated, PMNs were incubated for 5 minutes in buffer containing 0.3 mM HgCI_2_, followed by swelling in the presence of HgCI_2_ (LPS + HgCI_2_). After incubation for 5 minutes in 0.3 mM HgCI_2_, other PMNs were then incubated for 15 minutes in buffer containing 5 mM β-mercaptoethanol and swelling was monitored in the presence of β-mercaptoethanol (LPS + HgCI_2_ + β-merc). Cells were assumed to have a spherical shape with a diameter calculated as the average of the maximum and minimum dimensions of each cell. *P <0.05, ***P <0.001 from control by two-way analysis of variance for repeated measures followed by Bonferoni’s *post-hoc* test. **(B)** Microscopy photographs of the time course and osmotic swelling of PMNs (original magnification ×80).

We next examined whether the induction of Aqp1 water channel expression by LPS invoked functional changes in PMN plasma membrane permeability. As a surrogate for membrane permeability, we measured PMN relative volume V/Vo in serial micrographs in response to a hypotonic challenge, facilitated by lowering cell culture medium osmolarity from 300 to 150 mOsm/lt. This intervention was associated with a 1.8-fold rise in V/Vo, followed by return to the initial dimensions by 600 seconds (Figure 5). With LPS stimulation, however, the PMN V/Vo increase was more pronounced (2.2-fold) and did not return to the baseline value during the 600-second observation time (Figure 5, *P* <0.001) The LPS-triggered permeability response of PMN was inhibited by incubation with HgCl_2_ (0.3 mM for 5 minutes), which is known to block permeability of the Aqp1 water pore by forming a covalent attachment with cysteine 189 in the native molecule [16]. Furthermore, incubation for 15 minutes in 5 mM β-mercaptoethanol (Figure 5, three independent experiments, *P* <0.05), a reducer of protein disulfide bonds, abrogated the action of mercury inhibition on the mercury-sensitive residue of Aqp1 Cys^189^, and restored the effects of LPS.

## Discussion

Hospital-acquired sepsis is a major cause of mortality in critically ill patients. It is becoming increasingly evident that critical illness is associated with a state of functional immunosuppression [3,18,19], possibly following an acute phase of disproportionate immune system activation. This paradigm has triggered interest in mechanistic understanding of immune system (dys)function in sepsis. In this context, we initially undertook gene expression profiling in a small sample of polytrauma patients, who subsequently acquired sepsis, which returned a large set of genes with differential expression between patients who developed sepsis versus severe sepsis. Owing to the limited size of this pilot study, and the variability inherent to this methodology, we compared our dataset with published work in animal models of acute insults relevant to the ICU population, such as LPS-induced lung injury, a common organ failure in severe sepsis, using the ortholog gene approach. We were thus able to identify Aqp1 as a gene differentially expressed in experimental animals and humans on a consistent basis, indicating a potential involvement in the acute phase response. Importantly, Aqp1 upregulation seems to take place not only in lung tissue [20] but, as shown in our study, also in leukocytes, suggesting a potentially fundamental role in cellular adaptation to acute stress. Although Aqp1 can be found in various tissues and cell types, most notably red blood cells, renal tubules and endothelial cells [7,21-24], details on Aqp1 expression patterns and functional roles in leukocytes have not yet been described.

Aquaporins regulate cell membrane permeability in most cell types. Most isoforms are selective to water, although some, including aquaporin-7 and aquaporin-9, are permeable to other micromolecules, including urea and glycerol [25]. Leukocytes are known to express aquaporin-9, which is crucial to the formation of filopodia, finger-like membrane protrusions involved in cytokinesis [26,27]. We here report the inducible expression of Aqp1 in leukocytes in clinical sepsis and in response to LPS *in vitro*. This finding reveals a hitherto unknown and potentially fundamental function of this molecule in innate immunity.

To validate the results of the initial pilot gene expression study and meta-analysis, we prospectively followed Aqp1 expression in leukocytes of critically ill patients who were not septic upon ICU admission. We observed an increase in Aqp1 mRNA expression with the appearance of sepsis and septic shock. This effect was not dependent on the length of ICU stay, but was related to the septic process severity and strongly implies that Aqp1 is inducible in humans in the context of the immune response to infection.

Induction in Aqp1 was recently reported by Madonna and coworkers [28] in mouse hearts following septic endotoxemia. In contrast, Aqp1 was found downregulated in human pleural mesothelial cell lines (MET-5A) and primary rat lung microvessel endothelial cells following LPS administration [29,30], as well as in rat and murine models of LPS-induced lung injury [30,31]. In spite of these disparities, which may merely reflect tissue-specific effects of the molecule and differences in experimental design, the data support a novel role for this protein in the stress response to sepsis.

Since ICU-acquired sepsis is most commonly caused by LPS found in Gram-negative bacteria, we tested the role of LPS as an Aqp1 inducer in human PMNs. We found a statistically and biologically significant upregulation of Aqp1 by LPS. This was abrogated by pharmacological disruption of NF-κB signaling pathway, known to transduce signals downstream of the Toll-like-receptor 4, implying that LPS stimulates Aqp1 expression via NF-κB activation. LPS measurably stimulated PMNs, as indicated by a significant increase of IL-8 production and activation of NF-κB.

Placing naive cells in a hypoosmotic environment results in a time-dependent and HgCl_2_-sensitive increase in cell volume. This transient effect is followed by a gradual decline to the baseline values, possibly as a result of a compensatory escape of water and osmotically active molecules from the cell or derecruitment of Aqp1 from the cell membrane. In the presence of LPS, however, membrane permeability remained increased throughout the observation period (600 seconds), consistent with excess Aqp1.

The implications of LPS-induced cell membrane permeability on PMN function in sepsis are unknown. Besides permeability, however, Aqp1 could regulate the PMN stress response by other putative mechanisms. In this context, Aqp1 contributes to endothelial cell migration [32] by forming a critical scaffold for a plasma membrane-associated multiprotein complex important for cytoskeleton remodeling, adhesion and motility. Additionally, the formation of filopodia depends on assembly of an actin cytoskeletal scaffold to support the cell membrane protrusion. An early event in protrusion formation is a local increase in water permeability facilitated by aquaporins, which are thus critical to cell motility [33]. The theoretical significance of the herein demonstrated induction of Aqp1 may be to enhance the neutrophil’s ability to migrate to sites of infection. However, additional involvement in neutrophil activation (for example, phagocytosis and oxidative burst) and resistance to apoptosis [34] cannot be excluded.

Our study has the following limitations. The population of our initial study on gene expression profiling (that is, five polytrauma patients) was small due to limitation of funds. However, analysis identified genes, among them Aqp1, with statistically significant changes in their expression. Expression of Aqp1 in patients was probed in total leukocytes isolated from blood, whereas *in vitro* studies were performed on the PMN fraction, which contains mostly neutrophils. Nevertheless, it should be noted that leukocytes from septic patients are mainly neutrophils. Aside from this being one of the first reports of Aqp1 expression in human leukocytes and PMNs, our results document that sepsis and LPS are associated with Aqp1 induction in leukocytes and neutrophils and this could be an important event in neutrophil activation in the setting of sepsis, as well as a biomarker of the latter. Further studies on the regulation of the Aqp1 signal transduction pathway and functional significance may enhance our understanding of the host’s immune response, which appears crucial to the outcome of this devastating syndrome.

## Conclusions

Based on sepsis-specific overexpression, we propose that increased levels of leukocyte Aqp1 may play a role in sepsis. Aqp1 is upregulated in leukocytes of critically ill patients with hospital-acquired sepsis, while *in vitro* studies support triggering by LPS and transcriptional regulation by NF-κB. Aqp1 induction is moreover associated with increased cell membrane permeability and may therefore be of probable functional significance.

## Key messages

• Aqp1 is upregulated in leukocytes of critically ill patients with ICU-acquired sepsis.

• In PMNs from healthy donors stimulated with LPS, a statistically and biologically significant upregulation of Aqp1 is observed.

• By disrupting NF-κB signaling in PMNs, we found that Aqp1 was no longer upregulated, indicating that LPS can directly increase Aqp1 expression via NF-κB activation.

• Increased Aqp1 expression leads to increased PMN cell membrane permeability.

## Abbreviations

Aqp1: Aquaporin-1; bp: Base pairs; Cdc42: Cell division control protein 42 homolog; dNTP: Deoxynucleotide triphosphate; DTT: Dithiothreitol; ELISA: Enzyme-linked immunosorbent assay; GAPDH: Glyceraldehyde 3-phosphate dehydrogenase; IL: Interleukin; LPS: Lipopolysaccharide; NF: Nuclear factor; PBS: Phosphate-buffered saline; PCR: Polymerase chain reaction; PMN: Polymorphonuclear granulocyte.

## Competing interests

The authors declare that they have no competing interests.

## Authors’ contributions

AGV designed and conducted experiments and drafted the manuscript. NAM designed the study and drafted the manuscript. SEO designed and supervised the study and edited the manuscript. ZM collected and processed specimens and recorded patient data. EJ collected and processed specimens and recorded patient data. TP conducted the microarray experiments. AA edited the manuscript and provided valuable input to study design. CR edited the manuscript and provided valuable input to study design. VA designed the microarray study and edited the manuscript. AK designed the study, collected specimens, supervised patient enrollment, data collection and recording, and edited the manuscript. All authors read and approved the final manuscript.

## Supplementary Material

Additional file 1: Table S1 The following additional data are available with the online version of this paper. Additional file 1 presents the materials and methods of the gene expression profiling study in detail and the list of deregulated genes in leukocytes of trauma patients resulting from the gene expression profiling study (Additional file 1: Table S1). For the gene expression profiling study, blood samples of five polytauma, initially nonseptic patients were obtained upon admission to the ICU. A second blood sample was obtained from each patient within 48 hours of sepsis. Total RNA was isolated from leukocytes and hybridization was performed using Affymetrix® Human Hu133A 2.0 GeneChip™ arrays. Following validation of results, we selected the genes that were differentially expressed upon development of severe sepsis as compared with sepsis. Click here for file

## References

[B1] Annane D Bellissant E Cavaillon JM Septic shock Lancet 2005 17 63 78 10.1016/S0140-6736(04)17667-8 15639681

[B2] Dellinger RP Levy MM Rhodes A Annane D Gerlach H Opal SM Sevransky JE Sprung CL Douglas IS Jaeschke R Osborn TM Nunnally ME Townsend SR Reinhart K Kleinpell RM Angus DC Deutschman CS Machado FR Rubenfeld GD Webb S Beale RJ Vincent JL Moreno R Surviving Sepsis Campaign Guidelines Committee including The Pediatric Subgroup Surviving Sepsis Campaign: International Guidelines for Management of Severe Sepsis and Septic Shock, 2012 Intensive Care Med 2013 17 165 228 10.1007/s00134-012-2769-8 23361625 PMC7095153

[B3] Frazier WJ Hall MW Immunoparalysis and adverse outcomes from critical illness Pediatr Clin North Am 2008 17 647 668 xi 10.1016/j.pcl.2008.02.009 18501759 PMC2474674

[B4] American College of Chest Physicians/Society of Critical Care Medicine Consensus Conference Definitions for sepsis and organ failure and guidelines for the use of innovative therapies in sepsis Crit Care Med 1992 17 864 874 10.1097/00003246-199206000-00025 1597042

[B5] Agre P King LS Yasui M Guggino WB Ottersen OP Fujiyoshi Y Engel A Nielsen S Aquaporin water channels – from atomic structure to clinical medicine J Physiol 2002 17 3 16 10.1113/jphysiol.2002.020818 12096044 PMC2290382

[B6] Verkman AS Physiological importance of aquaporin water channels Ann Med 2002 17 192 200 12173689

[B7] Denker BM Smith BL Kuhajda FP Agre P Identification, purification, and partial characterization of a novel Mr 28,000 integral membrane protein from erythrocytes and renal tubules J Biol Chem 1988 17 15634 15642 3049610

[B8] Smith BL Agre P Erythrocyte Mr 28,000 transmembrane protein exists as a multisubunit oligomer similar to channel proteins J Biol Chem 1991 17 6407 6415 2007592

[B9] Papadopoulos MC Saadoun S Verkman AS Aquaporins and cell migration Pflugers Arch 2008 17 693 700 10.1007/s00424-007-0357-5 17968585 PMC3595095

[B10] Saadoun S Papadopoulos MC Davies DC Bell BA Krishna S Increased aquaporin 1 water channel expression in human brain tumours Br J Cancer 2002 17 621 623 10.1038/sj.bjc.6600512 12237771 PMC2364235

[B11] Loitto VM Forslund T Sundqvist T Magnusson KE Gustafsson M Neutrophil leukocyte motility requires directed water influx J Leukoc Biol 2002 17 212 222 11818441

[B12] Livak KJ Schmittgen TD Analysis of relative gene expression data using real-time quantitative PCR and the 2(−Delta Delta C(T)) method Methods 2001 17 402 408 10.1006/meth.2001.1262 11846609

[B13] Lleu PL Rebel G Interference of Good's buffers and other biological buffers with protein determination Anal Biochem 1991 17 215 218 10.1016/0003-2697(91)90210-K 2048724

[B14] Laemmli UK Cleavage of structural proteins during the assembly of the head of bacteriophage T4 Nature 1970 17 680 685 10.1038/227680a0 5432063

[B15] Batteiger B Newhall WJ Jones RB The use of Tween 20 as a blocking agent in the immunological detection of proteins transferred to nitrocellulose membranes J Immunol Methods 1982 17 297 307 10.1016/0022-1759(82)90089-8 6820029

[B16] Preston GM Jung JS Guggino WB Agre P The mercury-sensitive residue at cysteine 189 in the CHIP28 water channel J Biol Chem 1993 17 17 20 7677994

[B17] Bernard GR Artigas A Brigham KL Carlet J Falke K Hudson L Lamy M Legall JR Morris A Spragg R American–European Consensus Conference on ARDS Definitions, mechanisms, relevant outcomes, and clinical trial coordination Am J Respir Crit Care Med 1994 17 818 824 10.1164/ajrccm.149.3.7509706 7509706

[B18] Boomer JS To K Chang KC Takasu O Osborne DF Walton AH Bricker TL Jarman SD 2nd Kreisel D Krupnick AS Srivastava A Swanson PE Green JM Hotchkiss RS Immunosuppression in patients who die of sepsis and multiple organ failure JAMA 2011 17 2594 2605 10.1001/jama.2011.1829 22187279 PMC3361243

[B19] Menges T Engel J Welters I Wagner RM Little S Ruwoldt R Wollbrueck M Hempelmann G Changes in blood lymphocyte populations after multiple trauma: association with posttraumatic complications Crit Care Med 1999 17 733 740 10.1097/00003246-199904000-00026 10321662

[B20] Garcia JG Genomic investigations into acute inflammatory lung injury Proc Am Thorac Soc 2011 17 167 172 10.1513/pats.201101-002MS 21543796 PMC3131835

[B21] Bondy C Chin E Smith BL Preston GM Agre P Developmental gene expression and tissue distribution of the CHIP28 water-channel protein Proc Natl Acad Sci U S A 1993 17 4500 4504 10.1073/pnas.90.10.4500 8506291 PMC46539

[B22] Brown D Verbavatz JM Valenti G Lui B Sabolic I Localization of the CHIP28 water channel in reabsorptive segments of the rat male reproductive tract Eur J Cell Biol 1993 17 264 273 8223717

[B23] Hasegawa H Lian SC Finkbeiner WE Verkman AS Extrarenal tissue distribution of CHIP28 water channels by in situ hybridization and antibody staining Am J Physiol 1994 17 C893 C903 7513954 10.1152/ajpcell.1994.266.4.C893

[B24] Raina S Preston GM Guggino WB Agre P Molecular cloning and characterization of an aquaporin cDNA from salivary, lacrimal, and respiratory tissues J Biol Chem 1995 17 1908 1912 10.1074/jbc.270.4.1908 7530250

[B25] Gonen T Walz T The structure of aquaporins Q Rev Biophys 2006 17 361 396 10.1017/S0033583506004458 17156589

[B26] Karlsson T Glogauer M Ellen RP Loitto VM Magnusson KE Magalhaes MA Aquaporin 9 phosphorylation mediates membrane localization and neutrophil polarization J Leukoc Biol 2011 17 963 973 10.1189/jlb.0910540 21873454

[B27] Loitto VM Huang C Sigal YJ Jacobson K Filopodia are induced by aquaporin-9 expression Exp Cell Res 2007 17 1295 1306 10.1016/j.yexcr.2007.01.023 17346701

[B28] Madonna R Jiang J Geng YJ Attenuated expression of gelsolin in association with induction of aquaporin-1 and nitric oxide synthase in dysfunctional hearts of aging mice exposed to endotoxin Int J Immunopathol Pharmacol 2012 17 911 922 23298482 10.1177/039463201202500409

[B29] Liu L Xie C Effects of downregulation of aquaporin1 by peptidoglycan and lipopolysaccharide via MAPK pathways in MeT-5A cells Lung 2011 17 331 340 10.1007/s00408-011-9288-1 21647617

[B30] Xie YP Chen CP Wang JC Qian GS Wang YD Xiao ZL Experimental study on the expression and function of aquaporin-1 and aquaporin-5 in rats with acute lung injury induced by lipopolysaccharide Zhonghua Jie He He Hu Xi Za Zhi 2005 17 385 389 16008975

[B31] Su X Song Y Jiang J Bai C The role of aquaporin-1 (AQP1) expression in a murine model of lipopolysaccharide-induced acute lung injury Respir Physiol Neurobiol 2004 17 1 11 10.1016/j.resp.2004.05.001 15351300

[B32] Monzani E Bazzotti R Perego C La Porta CA AQP1 is not only a water channel: it contributes to cell migration through Lin7/beta-catenin PLoS One 2009 17 e6167 10.1371/journal.pone.0006167 19584911 PMC2701997

[B33] Loitto VM Karlsson T Magnusson KE Water flux in cell motility: expanding the mechanisms of membrane protrusion Cell Motil Cytoskeleton 2009 17 237 247 10.1002/cm.20357 19347962

[B34] Hoque MO Soria JC Woo J Lee T Lee J Jang SJ Upadhyay S Trink B Monitto C Desmaze C Mao L Sidransky D Moon C Aquaporin 1 is overexpressed in lung cancer and stimulates NIH-3T3 cell proliferation and anchorage-independent growth Am J Pathol 2006 17 1345 1353 10.2353/ajpath.2006.050596 16565507 PMC1606549

