# Supplementary material for: Induced expression and functional effects of aquaporin-1 in human leukocytes in sepsis
Source: Crit Care. 2013 Sep 12;17(5):R199. doi: 10.1186/cc12893 (PMC4056620; doi:10.1186/cc12893)
Supplement: Additional file 1: Table S1 — The following additional data are available with the online version of this paper. Additional file 1 presents the materials and methods of the gene expression profiling study in detail and the list of deregulated genes in leukocytes of trauma patients resulting from the gene expression profiling study (Additional file 1: Table S1). For the gene expression profiling study, blood samples of five polytauma, initially nonseptic patients were obtained upon admission to the ICU. A second blood sample was obtained from each patient within 48 hours of sepsis. Total RNA was isolated from leukocytes and hybridization was performed using Affymetrix® Human Hu133A 2.0 GeneChip™ arrays. Following validation of results, we selected the genes that were differentially expressed upon development of severe sepsis as compared with sepsis. [file cc12893-S1.doc]

Title: Induced expression and functional effects of Aquaporin-1 in human leukocytes in sepsis

**Authors**: Alice G. Vassiliou, PhD, Nikolaos A. Maniatis, MD, Stylianos E. Orfanos, MD, Zafeiria Mastora, MD, Edison Jahaj, BS, Triantafillos Paparountas, PhD, Apostolos Armaganidis, MD, Charis Roussos, MD/PhD, Vassilis Aidinis, PhD, Anastasia Kotanidou, MD.

**Materials and methods**

*Study population*

Blood samples of 5 polytauma, initially non-septic patients (one female) were obtained upon admission to the ICU of Evangelismos Hospital, Athens, Greece. A second blood sample was obtained from each patient within 48 hours of sepsis.

Patients were considered to have sepsis when they developed systemic inflammatory response syndrome as a result of documented infection, septic patients with evidence of organ dysfunction were considered to have severe sepsis, and septic patients with persisting hypotension (despite adequate fluid resuscitation) were considered to have septic shock, in accordance with international guidelines and recommendations [1, 2].

*RNA extraction and quality assessment*

Total RNA was isolated from total blood cells (>95% leukocytes) using the Trizol reagent (Invitrogen, Life Technologies Corporation, Carlsbad, CA, USA) according to manufacturer’s instructions. Following removal of short (<300nt) fragments through the use of RNeasy columns (QIAGEN GmbH, Hilden, Germany) as directed by the manufacturer, the RNA quantity and purity was assessed. Optical density measurements were taken at 260nm and 280nm using a Nanodrop spectrophotometer (Thermo Fisher Scientific Inc., Waltham, MA 02454, USA). The A260/A280 ratios for all samples ranged from 2.01-2.13, indicating good quality RNA. The Agilent Bioanalyzer (Agilent, Santa Clara, CA 95051, USA) was used for assessment of the RNA integrity.

*cRNA preparation for microarray hybridization*

From each sample, 1µg of total RNA was used to generate biotinylated complementary RNA (cRNA) according to the TWO-Cycle Target Labeling protocol (GeneChip™ Expression Analysis Technical Manual; Affymetrix, Santa Clara, CA 95051, USA; Revision 510). In short, biotinylated cRNA was synthesized using the GeneChip™ TWO-Cycle Target Labeling and Control Reagents (Affymetrix, Santa Clara, CA, USA), while cRNAs prior and following fragmentation [3] were analyzed by an Agilent 2100 Bioanalyzer. Fragmentation of cRNA target before hybridization onto GeneChip™ probe arrays has been shown to be critical in obtaining optimal assay sensitivity [3]. The Fragmentation Buffer has been optimized to break down full-length cRNA to 35 to 200 base fragments by metal-induced hydrolysis. The cRNA fragments are able to attach to the probe sequences found on the arrays surface with increased efficiency. Concentration of the newly generated cRNA samples were as expected for a successful processing [3] 10-times higher than the starting total RNA material.

*Test3 GeneChip™ microarray quality control hybridization*

To further determine the quality of each labeled target prior to its analysis with the Affymetrix® GeneChip™ expression arrays, the Affymetrix® GeneChip™ Test3 Array was utilized. Affymetrix® GeneChip™ Test3 Array contains probe sets representing a subset of characterized genes from various organisms, including mammals, plants, and eubacteria, while the signals obtained from these probe sets, allow the identification of degraded samples containing insufficient target that may result in poor expression array results. Additionally, GeneChip™ Test3 Array contains a subset of human and mouse housekeeping genes expressed early in fetal development and throughout adulthood. These genes may serve as a useful internal control [4].

Fragmented cRNA samples were first hybridized to the Test3 Array for 16 hours at 45°C, then washed and stained with streptavidin-phycoerythrin (SAPE) using the Affymetrix® GeneChip™ Fluidics Station 450 and images were acquired using Affymetrix® GeneChip Scanner 3000. Images were analyzed using Affymetrix® GeneChip™ Operating Software (GCOS) and comparison analysis was performed as directed by the manufacturer. All RNA samples analyzed by Test3 Array generated good RNA hybridization quality results.

*Affymetrix® Human Hu133A 2.0 GeneChip™ expression arrays hybridization*

This study utilized commercially available high-density Affymetrix®; Human Hu133A 2.0 microarrays, interrogating over 22,000 probe sets able to analyze the expression level of over 18,400 transcripts and variants, including more than 14,500 well-characterized human genes [5]. cRNA preparation, hybridization, and scanning of the arrays were performed according to the manufacturer’s protocols [3].

Fragmented cRNA (10μg) for each experimental sample was hybridized for 16 hours to the Affymetrix® Human Hu133A 2.0 GeneChip™ arrays, at 45°C in an Affymetrix® GeneChip™ Hybridization Oven 640. The Affymetrix® GeneChip™ Fluidics Station 450 was used to wash and stain the arrays with streptavidin-phycoerythrin, biotinylated anti-streptavidin according to the standard antibody amplification protocol for eukaryotic targets. For the aforementioned methods, the GeneChip™ Hybridization, Wash, and Stain Kits by Affymetrix® (Santa Clara, CA, USA) were used, while in parallel, the Affymetrix® eukaryotic hybridization control kit and Poly-A RNA control kit were used to ensure efficiency of hybridization and cRNA amplification. Arrays of samples were scanned with an Affymetrix® GeneChip™ Scanner 3000 at 570 nm and obtained images were visually screened to account for signal artifacts, scratches or debris. Images were analyzed using Affymetrix® GeneChip® Operating Software (GCOS) and comparison analysis was performed as directed by the manufacturer. All RNA samples analyzed by standard Array generated good RNA quality results. Probe intensities were extracted from the images using Affymetrix® software (Affymetrix GeneChip™ Operating Software; Affymetrix, Santa Clara, CA 95051, USA).

*Software quality controls*

Hybridization quality controls were performed using the Affymetrix® GeneChip™ Operating System (GCOS) indicating low background levels for all arrays (<84.94), acceptable range of present/absent calls for all probe sets 24.4%-40.2% Present calls) as well as for 3’/5’ fragment ratio of GAPDH and β-actin. Moreover, scaling factors for each array (calculated utilizing MAS5 software) were highly similar.

Raw data files were imported in:

i) R-Bioconductor v2.6.2 [6] and

ii) Partek® Genomics suite software ver6.3 (Partek Incorporated, St. Louis, MO, USA).

Array quality was tested with R-Bioconductor v2.6.2 libraries affyQCReport [7], affyPLM [8], affylmGUI [9] and oneChannelGUI [10]. Part of the quality control was done in R-Bioconductor v2.3.1 [6] using library AMDA [11] and with Partek® Genomics suite software ver6.3 (Partek Incorporated, St. Louis, MO, USA).

Present, Absent and Marginal calls, which **c**ategorize the summarized probe intensities of each separate array as “signal acceptable”, “signal not acceptable or absent”, and “marginal signal intensity” respectively were identified for each array probeset, utilizing the Affymetrix® MAS5 [3] algorithm implemented in the AMDA [11] and AFFY [12] library of R-Bioconductor [6].

*Normalization & statistical selection*

The perfect match (PM) raw intensities for each array were subsequently processed for PM signal noise correction, log2 base quantile normalization and median polish summarization utilising Partek® Genomics suite software ver6.3 (Partek Incorporated, St. Louis, MO, USA). Specifically, the RMA [13] algorithm with accounting for GC content was used while processing with Partek Genomics suite. Statistical analysis was performed utilizing Partek® Genomics suite software ver6.3 (Partek Incorporated, St. Louis, MO, USA), in order to select the differentially expressed genes. Due the experimental design, summary measures-differences between timepoints was the chosen method for statistical analysis.

Subsequently, statistical analysis using one-way ANOVA and Kruskal-Wallis were performed as appropriate to identify genes with statistically significant differences among the replicate values of -fold changes of the two separate patient groups, utilizing Partek® Genomics suite software ver6.3 (Partek Incorporated, St. Louis, MO, USA). The designated statistical selection cut-off points were: p value <0.05, FDR 5%. Statistical analysis yielded viable differentially expressed (1199 Unique ENTREZ GENE IDs = 1369 probesets) while (1091 unique ENTREZ GENE IDs = 1239 probesets) of those passed the ±1.5 fold change cutoff.

*Comparative analysis*

To enhance the validity of gene expression profiling comparative analysis was conducted for the genes found differentially expressed. Reported differentially expressed genes were retrieved from literature for LPS-induced and aseptic ALI [14-22]. All Identifiers were converted to Human Entrez Gene IDs by retrieving the orthologous genes through BioMart (access date 5th July 2008) [23].

## References

1. **American College of Chest Physicians/Society of Critical Care Medicine Consensus Conference: definitions for sepsis and organ failure and guidelines for the use of innovative therapies in sepsis**. *Crit Care Med* 1992, **20**(6):864-874.

2. Dellinger RP, Levy MM, Rhodes A, Annane D, Gerlach H, Opal SM, Sevransky JE, Sprung CL, Douglas IS, Jaeschke R *et al*: **Surviving Sepsis Campaign: International Guidelines for Management of Severe Sepsis and Septic Shock, 2012**. *Intensive Care Med* 2013, **39**(2):165-228.

3. Affymetrix: **Expression Analysis Technical Manual**. In*.*; 2004.

4. Affymetrix: **Affymetrix GeneChip® Test3 Arrays Package Inserts**. In*.*; 2003.

5. Affymetrix: **Technical Note: Design and Performance of the GeneChip®Human Genome U133 Plus 2.0 and Human Genome U133A 2.0 Arrays**. In*.*; 2004.

6. R Development Core Team: **R: A language and environment for statistical computing**. Vienna,Austria: R Foundation for Statistical Computing; 2008.

7. Parman C, Halling C: **affyQCReport: QC Report Generation for Affy Batch objects. R package version 2.6.2**. In*.*; 2008.

8. Bolstad B: **affyPLM:Methods for fitting probe-level models.R package version 2.6.2**. In*.*; 2008: A package that extends and improves the functionality of the base affy package. Routines that make heavy use of compiled code for speed. Central focus is on implementation of methods for fitting probe-level models and tools using these models. PLM based quality assessment tools.

9. Wettenhall J, Simpson K: **affylmGUI:GUI for affy analysis using limma package.R package version 2.6.2**. In*.*; 2008: A Graphical User Interface for affy analysis using the limma Microarray package.

10. Calogero RA: **oneChannelGUI:capabilities extension of affylmGUI graphical interface.R package version 2.6.2**. In*.* Torino, Italy; 2008.

11. Pelizzola M, Pavelka N, Foti M, Ricciardi-Castagnoli P: **AMDA: an R package for the automated microarray data analysis**. *BMC bioinformatics* 2006, **7**:335.

12. Irizarry RA, Gautier L, Bolstad BM, Miller C: **affy:Methods for Affymetrix Oligonucleotide Arrays.R package version 2.6.2**. In*.*; 2008.

13. Irizarry RA, Hobbs B, Collin F, Beazer-Barclay YD, Antonellis KJ, Scherf U, Speed TP: **Exploration, normalization, and summaries of high density oligonucleotide array probe level data**. *Biostatistics (Oxford, England)* 2003, **4**(2):249-264.

14. Copland IB, Kavanagh BP, Engelberts D, McKerlie C, Belik J, Post M: **Early changes in lung gene expression due to high tidal volume**. *Am J Respir Crit Care Med* 2003, **168**(9):1051-1059.

15. Dolinay T, Kaminski N, Felgendreher M, Kim HP, Reynolds P, Watkins SC, Karp D, Uhlig S, Choi AM: **Gene expression profiling of target genes in ventilator-induced lung injury**. *Physiological genomics* 2006, **26**(1):68-75.

16. Grigoryev DN, Ma SF, Irizarry RA, Ye SQ, Quackenbush J, Garcia JG: **Orthologous gene-expression profiling in multi-species models: search for candidate genes**. *Genome biology* 2004, **5**(5):R34.

17. Ma SF, Grigoryev DN, Taylor AD, Nonas S, Sammani S, Ye SQ, Garcia JG: **Bioinformatic identification of novel early stress response genes in rodent models of lung injury**. *Am J Physiol Lung Cell Mol Physiol* 2005, **289**(3):L468-477.

18. Jeyaseelan S, Chu HW, Young SK, Worthen GS: **Transcriptional profiling of lipopolysaccharide-induced acute lung injury**. *Infection and immunity* 2004, **72**(12):7247-7256.

19. Altemeier WA, Matute-Bello G, Gharib SA, Glenny RW, Martin TR, Liles WC: **Modulation of lipopolysaccharide-induced gene transcription and promotion of lung injury by mechanical ventilation**. *J Immunol* 2005, **175**(5):3369-3376.

20. Meyer NJ, Garcia JG: **Wading into the genomic pool to unravel acute lung injury genetics**. *Proc Am Thorac Soc* 2007, **4**(1):69-76.

21. dos Santos CC, Okutani D, Hu P, Han B, Crimi E, He X, Keshavjee S, Greenwood C, Slutsky AS, Zhang H *et al*: **Differential gene profiling in acute lung injury identifies injury-specific gene expression**. *Crit Care Med* 2008, **36**(3):855-865.

22. Gharib SA, Liles WC, Matute-Bello G, Glenny RW, Martin TR, Altemeier WA: **Computational identification of key biological modules and transcription factors in acute lung injury**. *Am J Respir Crit Care Med* 2006, **173**(6):653-658.

23. Haider S, Ballester B, Smedley D, Zhang J, Rice P, Kasprzyk A: **BioMart Central Portal--unified access to biological data**. *Nucleic acids research* 2009, **37**(Web Server issue):W23-27.

**Table 1:** Deregulated genes in leukocytes of trauma patients. Group 1, two patients who developed sepsis (defined as Systemic Inflammatory Response Syndrome and documented infection); group 2, three patients who developed severe sepsis with multi-organ dysfunction and Acute Respiratory Distress Syndrome (ARDS). Using ANOVA we detected genes differentially regulated among the two groups, which could be involved in pathogenetic mechanisms of the septic process. We found 1199 genes with statistically significant differences among the replicate values of -fold change of the two separate patient groups (p<0.05; FDR 5%).

*FC: fold change

Downregulated

Upregulated
